# Immune checkpoint inhibitor-related acral vasculitis

**DOI:** 10.1186/s40425-018-0443-6

**Published:** 2018-11-16

**Authors:** Thibault Comont, Vincent Sibaud, Loïc Mourey, Pierre Cougoul, Odile Beyne-Rauzy

**Affiliations:** 1 Service de Médecine Interne, Centre Hospitalier Universitaire Toulouse Purpan - Institut Universitaire du Cancer Toulouse Oncopole, 1 av Joliot-Curie, 31100 Toulouse, France; 2 Service d’Oncodermatologie, Institut Universitaire du Cancer Toulouse Oncopole, Toulouse, France; 3 Service d’Oncologie médicale, Institut Universitaire du Cancer Toulouse Oncopole, Toulouse, France

**Keywords:** Immune check point inhibitors, Immune-related adverse events, Vasculitis

## Abstract

Commentary on « Ipilimumab induced vasculitis » by Padda A. et al., J Immunother Cancer. 2018;6:12. The authors diagnosed a small vessel vasculitis following treatment with anti-CTLA-4 (ipilimumab) for a resected stage III B/C melanoma. We report a similar case of acral vasculitis occurring with a combination of anti-CTLA-4 (tremelimumab) and anti-PD-L1 (durvalumab) prescribed for the management of a metastatic urothelial bladder cancer. In contrast to Padda A. et al., we observed a significant improvement with oral corticosteroids.

## Main text

In the February 2018 edition of the Journal for ImmunoTherapy of Cancer, Padda A. et al. published an interesting case report of a 52-year old woman with a resected stage III B/C melanoma treated by high-dose of pilimumab (10 mg/kg) who developed severe digital ischemia [1]. Diagnosis of Ipilimumab-induced small vessel vasculitis was retained, requiring administration of high-dose corticosteroids, intravenous epoprostenol, botulinum toxin injections, and rituximab (weekly infusions, 375 mg/m2) for four cycles. The patient did not develop additional proximal digital ischemia but did require multiple distal digit amputations. We recently observed a similar case, following combined therapy with tremelimumab (antibody (ab) targeting cytotoxic T lymphocyte antigen 4 (CTLA-4)) and durvalumab (ab targeting Programmed death-ligand 1 (PD-L1)).

**Fig. 1:**
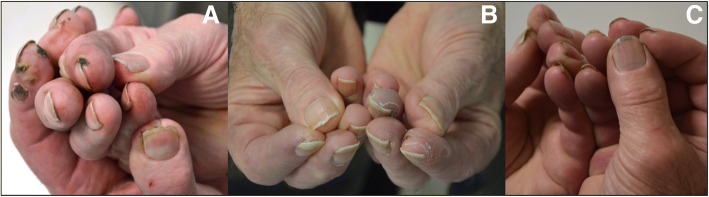
Digital lesions upon diagnosis of small vessel vasculitis (**a**), 1 month after initiation of corticosteroids (**b**) and at a one-year follow-up (**c**)

In 2012, a 66-year-old man was diagnosed with urothelial bladder cancer (stage III, pT2, high grade). He had hypertension (treated with calcium channel blockers) and history of smoking but no known history of cardiovascular disease, diabetes mellitus, autoimmune/rheumatologic or haematological disorders, no prior Raynaud’ s phenomenon or trauma. He was initially treated by chemotherapy (dose dense MVAC (methotrexate, vinblastine, doxorubicin and cisplatin)) and underwent surgery (radical cystoprostatectomy and ileal neobladder (Hartmann)). In 2015, he presented with anastomotic recurrence. An uretrectomy was performed and the Hartmann pouch was converted into a bricker ileal conduit. In May 2016, CT scan showed metastatic disease with bone and lymph nodes involvement. Starting September 2016, the patient was included in a clinical trial (NCT02516241) evaluating the efficacy and safety of the combination of tremelimumab (75 mg) and durvalumab (1500 mg) for 4 cycles, followed by durvalumab (1500 mg) monotherapy as maintenance. This treatment was initially well tolerated, without development of immune-related adverse events (IRAEs). In February 2017, slight erythema involving fingertips of both hands occurred, associated with paraesthesia and pain. Physical examination revealed grade 3 violaceous erythema involving all the fingers. Periungual skin necrosis of left hand (digits 2, 3 and 4) and right hand (digit 2) were also noted (*See* Fig. 1 a)*.* There were no clinical signs suggestive of associated rheumatologic or vascular diseases. Examination also revealed sensitive neuropathy on upper and lower limbs.

Immunologic tests, including protein electrophoresis, rheumatoid factor, cytoplasmic and perinuclear anti-neutrophil cytoplasmic ab, cryoglobulins, anti-phospholipids, anti-extractable nuclear antigen and anti-DNA ab were all negative, except for antinuclear ab, which were positive at 1:5200. Blood tests were also negative for hepatitis B/C, and haemostasis and thyroid parameters were within normal range. In addition, arterial Doppler of the upper limbs, cerebral MRI and echocardiography did not reveal any significant abnormalities. Periungual capillaroscopy showed peri-capillary oedema without any associated changes (including a lack of identified megacapillaries). Skin punch biopsy was performed on a perilesional area and did not reveal any pathological findings (epidermis and upper dermis were normal). However, biopsy was too superficial and did not include deep dermis and medium-sized vessels. Diagnosis of acral vasculitis was retained considering clinical and biological features. Spot urine test revealed no proteinuria. Electroneuromyography showed severe sensory-motor polyneuropathy. Prednisone was initiated (1 mg per kg administered daily over 15 days followed by progressive tapering) and immune checkpoint inhibitors were discontinued for a presumed tremelimumab and/or durvalumab IRAE. After 1 month of treatment, skin lesions partially improved (*See* Fig. 1 b)*.* Progressive recovery led to complete healing in 10 months, and corticosteroids were progressively tapered (*See* Fig. 1 c)*.* On the last follow-up (may 2018), no new metastases were detected and the disease was considered stable.

Immune checkpoint inhibitors (ICIs) are now approved by Food and Drug Administration and European Medicines Agency in a large range of advanced cancers. Although they present a favorable safety profile, IRAEs of any grade may occur in about 90 and 70% of patients treated in monotherapy with anti-CTLA-4 ipilimumab and with any anti-PD-1 or anti-PD-L1 antibody, respectively [2]. Moreover, IRAEs are more frequent, more severe and appear earlier when ICIs are used in combination. Vasculitis induced by ICIs is rare, and mainly involves large vessels (giant cell arteritis, isolated aortitis) or the nervous system (primary angiitis of the central nervous system and isolated vasculitis of the peripheral nervous system) [3]. Moreover, acral vasculitis has been exceptionally reported before Padda et al. report [1, 3–5] (Table 1). The first case was a male patient, treated with a combination of anti-PD-L1 therapy, BRAF inhibitor and MEK inhibitor for metastatic melanoma, who developed severe finger ischemia with necrosis associated with positive cryoglobulin and auto-SSA ab [4]. The second case was related to a paraneoplastic acral vascular syndrome in a patient with metastatic melanoma treated by a combination therapy of nivolumab (anti-PD1) and ipilimumab (anti-CTLA-4) without any detectable immunological changes [5]. In this latter case, authors discussed a paraneoplastic origin mediated by an immune mechanism. In our patient, histopathological analysis did not individualized vascular lesions. Typical clinical presentation, however, associated with a complete response with oral corticosteroids are clearly in favor of an ICI-related acral vasculitis.

**Table 1:** Previously published and current case of Immune checkpoint inhibitor-related acral vasculitis. Patient characteristics

	Age/Gender	Cancer	ICI	Onset*	Skin lesions	Systemic symptoms	Immunological findings	CTCAE	Treatment/outcome of the IRAE
Comont et al.	66/Male	Urothelial bladder cancer	Anti-CTLA4 (tremelimumab) Anti-PDL1 (durvalumab)	8	Periungual skin necrosis of several digits of both hands	None	ANAs (titer 5200, speckled pattern)	3	ICI discontinuation; Prednisone Complete resolution of IRAE
Padda er al.	52/Male	Melanoma	Anti-CTLA4 (ipilimumab)	4	Subungual necrosis on several upper and lower limb digits, rash	Myalgias, arthralgias, vision changes, jaw pain, interstitial pneumonia	None	3	ICI discontinuation; Methylprednisolone, prednisone, calcium channel blockers, nitropaste, poprostenol, botulinum toxin, sildenafil, rituximab Worsening of the IRAE requiring surgical amputation of multiple distal digits
Leburel et al.	60/Male	Melanoma	Anti-PDL1 (UKN) BRAF and MEK inhibitors (UKN)	8	Necrosis of 3 fingers and the heels	Arthralgia, dry mouth, paresthaesia of the feet and interstitial pneumonia	ANAs (titer 160, speckled pattern), Anti-SSA Abs Type III cryoglobulinemia	3	ICI discontinued; prednisone, calcium channel blockers, iloprost and acetylsalicylic acid Partial resolution of th.e IRAE
Gambichler et al.	60/Male	Melanoma	Anti-PD1 (nivolumab) Anti- CTLA4 (ipilimumab)	3	Subungual necrosis on the fingertips of both hands, severe gangrene	None	None	4	ICI discontinued after a second course of nivolumab; Prostacyclin, methylprednisolone Worsening of the IRAE requiring surgical amputation of multiple distal digits Progression of metastatic disease to multi-organ failure, leading to the death of the patient

*Weeks between initiation of immunotherapy and the diagnosis of vasculitis

*Ab* antibodies, *ANA* antinuclear antibody, *CTCAE* Common Terminology Criteria for Adverse Events, *ICI* immune checkpoint inhibitor, *IRAE* immune-related adverse event, *UKN* unknown

We describe here the first reported case of acral vasculitis induced after a combination therapy by tremelimumab and durvalumab. In contrast to other reports we observed complete resolution after a steroid-based treatment alone. This IRAE seems to be a class effect, which is probably more common with combination therapy.

Small vessel vasculitis with digital necrosis is now a known IRAEs which may occur with any ICI and requires close monitoring and early initiation of treatment to avoid extensive necrosis and other complications.

## Abbreviations

Ab: Antibody
CTLA-4: Cytotoxic T lymphocyte antigen 4
ICIs: Immune check point inhibitors
IRAEs: Immune related adverse events
PD-L1: Programmed death-ligand 1
